# The Ocular Surface Bacterial Microbiome and the Impact of Contact Lens Use: A Literature Review

**DOI:** 10.3390/microorganisms14030518

**Published:** 2026-02-24

**Authors:** Laura De Luca, Feliciana Menna, Stefano Lupo, Enzo Maria Vingolo, Matteo Mario Carlà, Maura Mancini, Giovanni William Oliverio, Letteria Minutoli, Antonio Baldascino, Cosimo Mazzotta, Pasquale Aragona, Alessandro Meduri

**Affiliations:** 1 Ophthalmology Clinic, Department of Biomedical Sciences, University of Messina, 98122 Messina, Italy; 2 Department of Medical-Surgical Sciences and Biotechnologies, U.O.C. Ophthalmology, Sapienza University of Rome, Via Firenze 1, 04019 Terracina, Italy; 3 Ophthalmology Department, “Fondazione Policlinico Universitario A. Gemelli, IRCCS”, 00168 Rome, Italy; 4 Faculty of Medicine and Surgery, University of Enna “Kore”, 94100 Enna, Italy; cgmazzotta@libero.it

**Keywords:** ocular surface microbiome, contact lens, microbial dysbiosis, contact lens wearer, literature review, microbiota

## Abstract

The ocular surface microbiome plays a critical role in maintaining ocular health, preventing infections, and regulating immune responses. Contact lens (CL) wear has been linked to alterations in microbial composition, potentially leading to dysbiosis and increased susceptibility to ocular infections. This review aims to summarize current evidence on the effects of CL use on the ocular microbiome and to discuss strategies to preserve microbial homeostasis. A literature search was conducted in PubMed, Scopus, Web of Science, and Google Scholar for English-language human studies published between January 2005 and January 2025. We included original studies and systematic reviews evaluating the ocular surface bacterial community in contact lens (CL) wearers using either sequencing-based approaches (microbiome; e.g., 16S rRNA gene sequencing/metagenomics) or culture-based methods (microbiota). Two authors screened titles/abstracts and full texts. Overall, 12 studies met the inclusion criteria and were qualitatively synthesized. Across included studies, CL wear was associated with reproducible changes in the ocular surface bacterial community, most commonly a shift toward a skin-like profile and increased detection/relative abundance of opportunistic taxa (e.g., *Pseudomonas*, *Acinetobacter*, and *Staphylococcus aureus*) together with reduced representation of typical ocular commensals in several sequencing-based datasets. Culture-based studies reported increased recovery of opportunistic bacteria from lenses and storage cases, supporting contamination/biofilm-related mechanisms. Lens care solutions and preservatives were reported to modulate bacterial profiles and may contribute to dysbiosis, although evidence remains heterogeneous across study designs and analytic pipelines. CL use is associated with significant alterations in the ocular microbiome, increasing the risk of microbial keratitis and corneal inflammatory events. Strategies to maintain microbial balance, including careful selection of lens care products and development of antimicrobial lenses, may improve ocular surface health in CL wearers. Future longitudinal studies with standardized sampling and analytic workflows are needed to clarify causal links between CL-associated microbial changes and clinical outcomes.

## 1. Introduction

The ocular surface (OS) is considered a functional unit whose role is the protection of the eye from environmental stress and form an ideal surface for corneal refraction through the creation of an efficient tear film [1]. The anatomical structures involved in this functional unit are the main lacrimal gland and the accessory lacrimal glands, which form the middle aqueous layer of the tear film, the conjunctival epithelium with its goblet cells, which form the inner mucous layer of the tear film, the Meibomian glands and the glands of Moll and Zeis, producing the outer lipid layer of the tear film, the corneal epithelium, and the nasolacrimal duct. The OS acts as an anatomical and functional unit able to maintain a controlled immunological reaction against antigenic challenges deriving from the external environment [2,3]. In healthy subjects without any OS disease an immunological activity is present supporting the role of the epithelia in providing an “immune tone” always existing in normal OS [4].

The presence in tears of antimicrobial components capable of inhibiting microbial survival and growth suggests that the ocular surface possesses a specific microbiome, similarly to other human sites such as the gut, skin, vagina, oral and nasal cavities. A microbiome has been defined “a characteristic microbial community occupying a reasonably well-defined habitat which has distinct physio-chemical properties” [5]. Importantly, in this review we use the term “microbiome” to indicate sequencing-based, DNA-level characterization of microbial communities (e.g., 16S rRNA gene sequencing, metagenomics), whereas we use “microbiota” to refer to culture-based detection of microorganisms. Because both approaches are represented in the CL literature, we report and interpret sequencing-based and culture-based evidence separately to avoid conflating community profiling with isolate recovery. However, the OS microbiome was not included in the Human Microbiome Project (2012) and in the Integrative Human Microbiome Project (2019), probably owing to its low biomass evidence. In fact, when compared to adjacent skin or other mucous membranes, healthy OS microbiome is paucibacterial, being 150 to 200 times fewer bacterial cells than the adjacent facial skin [6], and its components are different from adjacent skin. The OS epithelium can detect highly conserved pathogen-associated membrane patterns (PAMPs) present on microbes by Toll-like receptors (TLRs).

Furthermore, particularly different resident microbial communities occupy various regions or microhabitats on the OS, thus changing the composition of the ocular microflora [7]. Differences have been confirmed, especially between the lid margins or skin, which has the highest abundance of bacteria, and the bulbar conjunctiva, with the lowest concentration. In recent years, a large number of papers have demonstrated the key role of the ocular microbiome in maintaining a physiological environment and, when altered, its correlation with OS disorders. The human ocular surface microbiome is a critical component of ocular health, playing a protective role against pathogenic invasions.

Unlike the gut microbiome, which has been extensively studied, the ocular microbiome remains less understood due to its lower microbial load and continuous exposure to antimicrobial factors in tears. Advances in metagenomics and next-generation sequencing have allowed for deeper exploration of the microbial communities inhabiting the ocular surface, revealing significant interactions between microbiota, ocular immunity, and external influences such as CL and pharmacological treatments.

The aims of this literature review were: (i) to summarize current evidence on how contact lens wear influences the ocular surface bacterial community; (ii) to distinguish findings derived from sequencing-based microbiome studies from those derived from culture-based microbiota studies; and (iii) to discuss the potential clinical relevance of these changes (e.g., corneal inflammatory events and microbial keratitis) and preventive/mitigating strategies, including lens care practices and emerging antimicrobial lens technologies.

## 2. Materials and Methods

A comprehensive bibliographic search was conducted in PubMed, Scopus, Web of Science, and Google Scholar to identify relevant studies published between January 2005 and January 2025. The search strategy included combinations of the following terms: (microbiome OR microbiota OR dysbiosis) AND (eye OR ocular surface OR cornea OR conjunctiva) AND (contact lens OR keratitis OR biofilm), and was restricted to human studies published in English. Studies were categorized a priori as sequencing-based microbiome studies or culture-based microbiota studies, and results were synthesized in separate subsections accordingly. This approach was adopted to maintain conceptual consistency between community-level profiling and culture-based isolate recovery.

After duplicate removal, titles and abstracts were independently screened by two authors. Full-text articles were subsequently assessed for eligibility, and any disagreements regarding study inclusion were resolved through consultation with a third author. Studies were excluded during screening if they were not related to CL wear, did not evaluate the ocular surface microbiome/microbiota, were non-human studies, were case reports, were conference abstracts, or were non-English publications. During full-text assessment, studies were excluded if they lacked microbiome analysis (e.g., clinical infection reports without microbial community assessment), did not include CL-related comparisons, or did not provide sufficient methodological details regarding microbial detection. Ultimately, 12 studies met the inclusion criteria and were included in the qualitative synthesis. A structured qualitative synthesis was performed, emphasizing study design, sampling site (conjunctiva/tear film/CL/lens case), microbial detection method, and the direction of reported changes in dominant taxa or isolate recovery.

Given the heterogeneity of study designs, populations, and microbiome analytical pipelines, a formal quantitative risk-of-bias assessment tool was not applied. Instead, a narrative methodological appraisal was conducted. For each included study, the following items were extracted and evaluated: study design, sample size, participant characteristics, sampling site (conjunctiva, tear film, CL, or lens case), CL modality and material (when available), sequencing or molecular method (e.g., 16S rRNA gene sequencing or culture-based methods), bioinformatic or analytic approach (when reported), and the presence of statistical testing for group comparisons. These elements were used to contextualize the strength and comparability of the available evidence.

Data extraction was performed independently by two authors using a predefined extraction grid. Extracted variables included year of publication, study setting, study design, sample size, participant status (healthy subjects vs. CL wearers), CL type (soft, orthokeratology, or scleral), exposure to care solutions (when applicable), sampling site, microbiome assessment method, main taxa reported as differentially abundant, and principal clinical associations (e.g., discomfort, corneal infiltrative events, or keratitis). Owing to methodological heterogeneity across studies, results were synthesized qualitatively using a narrative approach rather than meta-analysis.

Although 12 studies met the inclusion criteria, Table 1 summarizes 10 representative studies selected to provide a concise overview of the most relevant and methodologically informative evidence. MeSH-based searches, gray literature screening, and manual hand-searching of reference lists were not systematically performed and represent limitations of the present review.

## 3. Results

In the following sections, we report findings from sequencing-based microbiome studies separately from culture-based microbiota studies to ensure consistency in interpretation.

### 3.1. Baseline Ocular Surface Bacterial Community in Healthy Subjects

Sequencing-based and culture-based studies have identified a limited but consistent bacterial presence on the healthy ocular surface. Commonly reported genera include *Staphylococcus*, *Corynebacterium*, *Streptococcus*, and *Propionibacterium* [18,19]. The ocular surface is characterized by multiple antimicrobial defense mechanisms, and some authors have described it as a low-biomass environment with features approaching near-sterility [18].

Tear film components, including lactoferrin, lysozyme, secretory immunoglobulin A, secretory phospholipase A2, and complement, have been identified as contributing to microbial regulation on the ocular surface [19]. In a clinical study evaluating liposomal lactoferrin eye drops, a reduction in the proportion of potentially pathogenic bacteria was observed after treatment compared with baseline, while the physiological microbial composition remained unchanged [20,21].

Alterations in ocular surface bacterial profiles have also been reported in association with topical medications and systemic conditions. In a study of patients treated with benzalkonium chloride–preserved glaucoma medications, Chang et al. (2022) reported increased bacterial diversity and a higher relative abundance of Gram-negative organisms compared with controls [8].

Systemic metabolic modulation has also been associated with ocular surface microbial changes. Wang et al. (2023) reported increased detection of *Bacteroides*, *Faecalibacterium*, and other taxa following canagliflozin treatment in patients with type 2 diabetes mellitus, together with a reduction in *Acinetobacter* species [22].

In HIV-infected individuals, Liu et al. reported differences in conjunctival bacterial composition compared with HIV-negative controls, with increased representation of Proteobacteria and Bacteroidetes and reduced Actinobacteria and Firmicutes in both untreated and treated HIV-positive groups [23].

Repeated antibiotic exposure has been associated with changes in conjunctival bacterial profiles. Studies evaluating topical azithromycin and fluoroquinolone use reported increased isolation of *Staphylococcus epidermidis* and *Staphylococcus aureus*, together with reduced recovery of Gram-negative species in fluoroquinolone-treated eyes [23,24].

In a cluster-randomized trial evaluating repeated mass azithromycin administration, Doan et al. (2020) identified *Haemophilus*, *Moraxella*, *Lactobacillus*, and *Streptococcus* as predominant genera on the ocular surface, with significant community-level changes after repeated treatment cycles [25].

In patients with keratitis, metagenomic analyses demonstrated increased representation of *Pseudomonas* species compared with healthy controls, including taxa harboring multidrug-resistance–associated genes [26].

### 3.2. Contact Lens Wear and Ocular Surface Microbiome Changes (Sequencing-Based Evidence)

Sequencing-based studies consistently assessed ocular surface bacterial community composition using 16S rRNA gene sequencing or related analytic pipelines, reporting relative abundance patterns and diversity metrics.

Across multiple studies, CL wear was associated with a recurrent shift toward a skin-like bacterial signature and higher representation of opportunistic taxa, although the magnitude of change varied by sampling site and analytic workflow [9,10,11,27].

Shin et al. (2016) reported that CL wearers exhibited increased relative abundances of *Methylobacterium*, *Acinetobacter*, and *Pseudomonas*, together with reduced representation of *Haemophilus*, *Streptococcus*, and *Corynebacterium* [9].

Retuerto et al. (2019) identified a higher relative abundance of Gram-negative bacteria, particularly *Pseudomonas* and *Acinetobacter*, in bacterial communities associated with worn contact lenses compared with non–CL wearers [10].

Sequencing-based studies reported similar patterns across different CL modalities. Soft contact lenses and orthokeratology lenses were both associated with increased detection of opportunistic taxa, including *Staphylococcus aureus* and *Corynebacterium* [11,27].

Several studies reported increased microbial diversity in CL wearers compared with non-wearers, together with detection of environmental, commensal, and potentially pathogenic bacteria on worn lenses and lens-associated samples [9,10].

Lens care practices were also evaluated in sequencing-based analyses. Hotta et al. (2020) reported that specific storage solutions were associated with selective enrichment of Streptococcus and *Staphylococcus* species in tear fluid and lens-associated samples [12].

Comparative analyses of symptomatic and asymptomatic CL wearers showed differences in bacterial profiles across conjunctival, lens, and lens-case samples. Raksha et al. (2019) reported higher representation of *Cutibacterium* and *Pseudomonas* in symptomatic wearers [13].

In scleral lens wearers, Dogan et al. (2020) identified increased detection of *Haemophilus* and *Moraxella* species [14].

Associations between microbial profiles and corneal infiltrative events have also been reported. Sankaridurg et al. (2000) demonstrated higher Gram-negative bacterial colonization of soft contact lenses during corneal infiltrative episodes compared with asymptomatic wear [15].

In a randomized controlled trial, Kalaiselvan et al. (2022) reported that antimicrobial peptide–coated contact lenses did not alter conjunctival microbiota composition while reducing bacterial colonization on the lens surface compared with uncoated lenses [16].

Chao et al. (2018) reported an association between lower microbial diversity and increased risk of corneal infiltrative events in CL wearers [17].

### 3.3. Contact Lens Wear and Ocular Surface Microbiota Findings (Culture-Based Evidence)

Culture-based studies evaluated bacterial growth from conjunctival swabs, worn contact lenses, and lens storage cases, thereby providing information on cultivable microorganisms rather than community-wide bacterial structure. These studies primarily reported the presence, frequency, and relative recovery of specific bacterial species.

Across multiple investigations, culture-based analyses consistently demonstrated higher bacterial recovery from worn contact lenses and lens cases compared with conjunctival samples. Frequently isolated organisms included *Staphylococcus* spp., *Pseudomonas* spp., *Acinetobacter* spp., and other Gram-negative bacteria [10,13,14,15].

Retuerto et al. (2019) reported increased isolation of Gram-negative bacteria, particularly *Pseudomonas* and *Acinetobacter*, from worn soft contact lenses and lens cases compared with samples from non–contact lens wearers [10]. Similarly, Raksha et al. (2019) identified differences in bacterial isolates recovered from the conjunctiva, contact lenses, and storage cases of symptomatic versus asymptomatic contact lens users, with higher recovery of *Pseudomonas* and *Cutibacterium* species in symptomatic individuals [13].

Studies focusing on specific contact lens modalities reported comparable findings. In scleral lens wearers, Dogan et al. (2020) identified increased recovery of *Haemophilus* and *Moraxella* species from conjunctival samples compared with non–lens-wearing controls [14].

Culture-based evidence has also highlighted associations between bacterial colonization of contact lenses and corneal infiltrative events. Sankaridurg et al. (2000) demonstrated that soft contact lenses worn during corneal infiltrative episodes showed significantly higher levels of Gram-negative bacterial colonization than lenses worn during asymptomatic periods [15].

Overall, culture-based studies indicate that contact lens wear is associated with increased recovery of opportunistic and potentially pathogenic bacteria from lenses and lens-related accessories, while conjunctival samples generally show lower bacterial yields.

### 3.4. Lens Care Solutions, Preservatives, and Other Exposures

Both sequencing-based and culture-based studies have evaluated the influence of lens care solutions, preservatives, and topical/systemic exposures on ocular surface bacterial profiles. These investigations primarily assessed changes in bacterial recovery, relative abundance, or diversity in relation to specific chemical or pharmacological exposures.

Hotta et al. (2020) analyzed bacterial profiles associated with contact lens care solutions and tear fluids and reported selective enrichment of *Streptococcus* and *Staphylococcus* species in association with certain multipurpose storage solutions [12]. Culture-based analyses also demonstrated that lens cases and solutions can serve as reservoirs for bacterial growth, with recovery of Gram-positive and Gram-negative organisms varying according to solution type and usage patterns [10,13].

Topical ophthalmic medications containing preservatives have been associated with changes in ocular surface bacterial profiles. In a case–control study, Chang et al. (2022) reported increased bacterial diversity and a higher relative abundance of Gram-negative organisms in patients treated with benzalkonium chloride–preserved glaucoma medications compared with controls [8].

Systemic pharmacological exposures have also been examined. Wang et al. (2023) reported alterations in ocular surface bacterial taxa following treatment with canagliflozin in patients with type 2 diabetes mellitus, including increased detection of taxa also present in the gut microbiota and reduced representation of *Acinetobacter* species [22].

Antibiotic exposure has been evaluated in both topical and systemic contexts. Studies assessing repeated topical antibiotic use reported increased recovery of *Staphylococcus epidermidis* and *Staphylococcus aureus*, together with reduced isolation of Gram-negative species in fluoroquinolone-treated eyes [23,24]. In a cluster-randomized trial, Doan et al. (2020) reported significant changes in ocular surface bacterial composition following repeated mass azithromycin administration, as assessed by metagenomic RNA sequencing [25].

Collectively, these studies demonstrate that lens care products, preservatives, and pharmacological exposures are associated with measurable changes in ocular surface bacterial profiles, as detected by both culture-based and sequencing-based methodologies.

### 3.5. Emerging Strategies

Pharmacological exposures, including topical and systemic antibiotics, have been associated with changes in ocular surface bacterial profiles, including increased recovery of resistant strains and altered relative abundance patterns [14,28].

Emerging strategies such as antimicrobial peptide–coated contact lenses have been evaluated in controlled clinical settings. Kalaiselvan et al. (2022) reported reduced bacterial colonization on peptide-coated lenses without detectable alterations in conjunctival microbiota composition [16].

## 4. Discussion

The ocular surface microbiome represents a low-biomass yet tightly regulated microbial ecosystem that plays a crucial role in maintaining ocular surface homeostasis. Although historically considered nearly sterile, advances in culture-independent sequencing techniques have demonstrated the presence of a consistent conjunctival bacterial community whose composition may be influenced by external factors such as CL wear, lens care solutions, and pharmacological exposures. The present review synthesizes available evidence indicating that these factors are associated with measurable alterations in ocular surface bacterial profiles and may contribute to dysbiosis, potentially increasing susceptibility to ocular surface inflammation and infection. It should be noted, however, that current microbiome-based evidence predominantly reflects bacterial components, as data on fungal, viral, and protozoal communities in CL wearers remain limited.

### 4.1. Potential Mechanisms Underlying Contact Lens–Associated Microbial Changes

Several non-mutually exclusive mechanisms may contribute to the microbial alterations observed in CL wearers. The presence of a contact lens can modify tear film structure and turnover, alter local oxygen availability, and change the epithelial microenvironment, thereby creating conditions that may favor bacterial adhesion and persistence. These environmental modifications have been consistently reported across different CL modalities and sampling sites [9,10,11].

Repeated hand–eye contact during lens insertion and removal may further facilitate the transfer of skin-associated microorganisms to the ocular surface and lens. This mechanism is consistent with sequencing-based findings showing a recurrent shift toward a skin-like bacterial profile in CL wearers, characterized by increased relative abundances of taxa such as *Methylobacterium*, *Acinetobacter*, and *Pseudomonas*, together with reduced representation of typical ocular commensals [9,10,11].

Lens material properties, surface characteristics, and oxygen permeability may additionally influence bacterial adhesion and biofilm formation, potentially modulating the magnitude of microbial changes. In scleral lens wear, the presence of a tear reservoir and reduced tear exchange may further promote microbial persistence on the ocular surface, contributing to distinct bacterial patterns reported in this subgroup [14].

### 4.2. Clinical Relevance of Microbiome and Microbiota Alterations in Contact Lens Wearers

From a clinical perspective, CL-associated enrichment of opportunistic taxa and increased bacterial recovery from lenses and lens cases may be relevant to the development of corneal infiltrative events and microbial keratitis. Several studies have reported associations between reduced microbial diversity, increased Gram-negative colonization, and corneal inflammatory events in CL wearers [15,17]. Increased bacterial colonization of soft contact lenses, particularly by Gram-negative organisms, has been observed during corneal infiltrative episodes compared with asymptomatic wear [15].

Pharmacological exposures represent an additional clinically relevant factor. Repeated use of topical antibiotics has been associated with altered conjunctival bacterial profiles and increased recovery of potentially resistant organisms, including *Staphylococcus epidermidis* and *Staphylococcus aureus* [23,26]. Similarly, preserved ophthalmic medications, particularly those containing benzalkonium chloride, have been linked to increased bacterial diversity and a relative predominance of Gram-negative organisms on the ocular surface [21].

However, it is important to emphasize that most available studies are cross-sectional and observational in nature, limiting causal inference. Microbiome and microbiota findings should therefore be interpreted as associative signals rather than direct predictors of disease. Not all investigations have reported marked differences between CL users and non-users, and some studies suggest that overall conjunctival microbial profiles may remain relatively stable despite the presence of keratitis-associated taxa in both groups [29].

### 4.3. Methodological Considerations and Future Directions

Interpretation of ocular surface microbiome data is challenged by several methodological limitations. The ocular surface is a low-biomass environment, which increases susceptibility to contamination and batch effects in sequencing workflows. Differences in sampling site (conjunctiva, tear film, contact lens, or lens case), sequencing regions and primers, use of negative controls, and bioinformatic pipelines substantially limit comparability across studies.

Culture-based investigations, while clinically meaningful, capture only cultivable organisms and do not reflect full community structure. Conversely, sequencing-based approaches provide broader community-level insights but may overestimate diversity or relative abundance in low-biomass samples if stringent contamination controls are not applied.

Additional heterogeneity arises from differences in study populations, lens modalities, hygiene practices, duration of CL wear, and exposure to lens care solutions or topical medications. These factors likely contribute to discrepant findings across studies and underscore the need for standardized methodologies.

Preventive strategies aimed at preserving ocular surface microbial homeostasis remain essential. Proper lens hygiene, avoidance of water exposure during CL wear, and careful selection of lens care solutions are critical components of risk reduction. Emerging approaches, such as antimicrobial peptide-coated contact lenses, have demonstrated reduced bacterial colonization without significant alteration of conjunctival microbial composition, offering a promising targeted strategy to mitigate infection risk while preserving the physiological microbiome [16].

Despite growing interest in this field, important gaps remain in understanding the temporal dynamics of ocular microbiome changes and their direct relationship with clinical outcomes. Well-powered longitudinal studies employing standardized sampling, sequencing, and analytic protocols are needed to identify reproducible microbial signatures and to guide the development of evidence-based preventive and therapeutic strategies for CL wearers.

## 5. Conclusions

The interplay between CL wear and the ocular microbiome is complex, involving shifts in microbial composition that can predispose individuals to infections. While CLs offer significant benefits for vision correction, they also pose challenges related to microbial contamination and ocular health. Pharmacological interventions, such as antibiotics, must be used judiciously to avoid disrupting the delicate microbial balance. Innovations like antimicrobial-coated lenses show promise in reducing infection risks without adversely affecting the microbiome. Overall, sequencing-based microbiome studies and culture-based microbiota studies converge in suggesting that CL wear is associated with measurable bacterial changes; however, the two approaches provide different levels of inference and should not be interpreted interchangeably. Ultimately, comprehensive education on proper CL hygiene and awareness of potential risks are essential in promoting ocular health and preventing adverse outcomes among CL users.

## Figures and Tables

**Table 1:** Different relevant clinical studies for the impact of contact lens wear and medications on ocular surface microbiome.

Study	Study Design	Sample Size	Contact Lens/Exposure Type	Microbial Detection Method	Sampling Site	Main Microbiome/Microbiota Findings	Clinical Implications
Chang et al. (2022) [8]	Case–control	40 subjects	Benzalkonium chloride–preserved glaucoma drops	Sequencing-based microbiome (16S rRNA)	Conjunctiva	Increased bacterial diversity and Gram-negative predominance	Preservatives influence ocular surface microbiome
Shin et al. (2016) [9]	Cross-sectional	58 subjects	Soft contact lenses	Sequencing-based microbiome (16S rRNA gene sequencing)	Conjunctiva; Contact lens	Shift toward skin-like microbiome with increased *Methylobacterium*, *Acinetobacter*, *Pseudomonas* and reduced ocular commensals	CL wear alters ocular bacterial community structure
Retuerto et al. (2019) [10]	Observational	59 lenses/cases	Worn soft contact lenses	Sequencing-based microbiome (16S rRNA) + culture-based microbiota	Contact lens; Lens case	Higher abundance of Gram-negative bacteria, particularly *Pseudomonas* and *Acinetobacter*	Highlights role of lenses and cases as microbial reservoirs
Zhang et al. (2017) [11]	Cross-sectional	30 subjects	Soft CL vs. orthokeratology	Sequencing-based microbiome (16S rRNA)	Conjunctiva	Increased opportunistic taxa (*Staphylococcus aureus*, *Corynebacterium*) in both CL types	Lens wear–associated changes independent of modality
Hotta et al. (2020) [12]	Observational	20 subjects	CL care solutions	Sequencing-based microbiome (16S rRNA) + culture-based microbiota	Tear film; Contact lens; Lens case	Selective enrichment of *Streptococcus* and *Staphylococcus* species	Care solutions modulate microbial composition
Raksha et al. (2019) [13]	Observational	40 subjects	Symptomatic vs. asymptomatic CL users	Culture-based microbiota	Conjunctiva; Contact lens; Lens case	Higher recovery of *Cutibacterium* and *Pseudomonas* in symptomatic users	Association with inflammatory symptoms
Dogan et al. (2020) [14]	Cross-sectional	18 subjects	Scleral lenses	Culture-based microbiota	Conjunctiva	Increased recovery of *Haemophilus* and *Moraxella*	Lens design may influence microbial persistence
Sankaridurg et al. (2000) [15]	Case–control	87 lenses	Soft CL during CIEs	Culture-based microbiota	Contact lens	Increased Gram-negative colonization during corneal infiltrative events	Lens colonization linked to CIE risk
Kalaiselvan et al. (2022) [16]	Randomized controlled trial	176 eyes	Antimicrobial peptide-coated CLs	Culture-based microbiota	Conjunctiva; Contact lens	Reduced lens colonization without changes in conjunctival microbiota	Antimicrobial lenses may reduce infection risk
Chao et al. (2018) [17]	Case–control	62 subjects	Soft CL users	Sequencing-based microbiome (16S rRNA)	Conjunctiva	Lower microbial diversity associated with corneal infiltrates	Microbiome diversity linked to inflammatory events

## Data Availability

No new data were created or analyzed in this study. Data sharing is not applicable to this article.
